# IEAtlas: an atlas of HLA-presented immune epitopes derived from non-coding regions

**DOI:** 10.1093/nar/gkac776

**Published:** 2022-09-13

**Authors:** Yangyang Cai, Dezhong Lv, Donghao Li, Jiaqi Yin, Yingying Ma, Ya Luo, Limei Fu, Na Ding, Yongsheng Li, Zhenwei Pan, Xia Li, Juan Xu

**Affiliations:** College of Bioinformatics Science and Technology, Harbin Medical University, Harbin, Heilongjiang 150081, China; College of Bioinformatics Science and Technology, Harbin Medical University, Harbin, Heilongjiang 150081, China; College of Bioinformatics Science and Technology, Harbin Medical University, Harbin, Heilongjiang 150081, China; College of Bioinformatics Science and Technology, Harbin Medical University, Harbin, Heilongjiang 150081, China; College of Bioinformatics Science and Technology, Harbin Medical University, Harbin, Heilongjiang 150081, China; College of Bioinformatics Science and Technology, Harbin Medical University, Harbin, Heilongjiang 150081, China; College of Bioinformatics Science and Technology, Harbin Medical University, Harbin, Heilongjiang 150081, China; College of Bioinformatics Science and Technology, Harbin Medical University, Harbin, Heilongjiang 150081, China; Key Laboratory of Tropical Translational Medicine of Ministry of Education, College of Biomedical Information and Engineering, Hainan Women and Children's Medical Center, Hainan Medical University, Haikou, Hainan 571199, China; Department of Pharmacology (State-Province Key Laboratories of Biomedicine-Pharmaceutics of China, Key Laboratory of Cardiovascular Research, Ministry of Education), College of Pharmacy, Harbin Medical University, Harbin, Heilongjiang 150086, China; NHC Key Laboratory of Cell Transplantation, The First Affiliated Hospital of Harbin Medical University, Harbin, Heilongjiang 150001, China; College of Bioinformatics Science and Technology, Harbin Medical University, Harbin, Heilongjiang 150081, China; Key Laboratory of Tropical Translational Medicine of Ministry of Education, College of Biomedical Information and Engineering, Hainan Women and Children's Medical Center, Hainan Medical University, Haikou, Hainan 571199, China; College of Bioinformatics Science and Technology, Harbin Medical University, Harbin, Heilongjiang 150081, China

## Abstract

Cancer-related epitopes can engage the immune system against tumor cells, thus exploring epitopes derived from non-coding regions is emerging as a fascinating field in cancer immunotherapies. Here, we described a database, IEAtlas (http://bio-bigdata.hrbmu.edu.cn/IEAtlas), which aims to provide and visualize the comprehensive atlas of human leukocyte antigen (HLA)-presented immunogenic epitopes derived from non-coding regions. IEAtlas reanalyzed publicly available mass spectrometry-based HLA immunopeptidome datasets against our integrated benchmarked non-canonical open reading frame information. The current IEAtlas identified 245 870 non-canonical epitopes binding to HLA-I/II allotypes across 15 cancer types and 30 non-cancerous tissues, greatly expanding the cancer immunopeptidome. IEAtlas further evaluates the immunogenicity via several commonly used immunogenic features, including HLA binding affinity, stability and T-cell receptor recognition. In addition, IEAtlas provides the biochemical properties of epitopes as well as the clinical relevance of corresponding genes across major cancer types and normal tissues. Several flexible tools were also developed to aid retrieval and to analyze the epitopes derived from non-coding regions. Overall, IEAtlas will serve as a valuable resource for investigating the immunogenic capacity of non-canonical epitopes and the potential as therapeutic cancer vaccines.

## INTRODUCTION

T-cell based cancer immunotherapies engage the immune system to recognize and eliminate tumor cells, and the recognition of tumor cells by natural killer T cells relies on presentation of tumor peptides by human leukocyte antigen (HLA) molecules. HLA-bound peptide sequences on the target cell surface are usually called ‘epitopes’, while their parent proteins are referred to as ‘antigens’ (1). There are two major types of HLA molecules. HLA class I (HLA-I) molecules present epitopes 8–12 amino acid long derived mainly from a variety of cytoplasmatic proteins and interact with CD8+ T cells. In contrast, HLA class II (HLA-II) molecules present longer peptides and interact with CD4+ T cells, which are predominantly sampled from endosomal and ingested proteins (2). Previous studies have widely explored canonical tumor antigens for cancer immunotherapy, which are encoded within the open reading frames (ORFs) of protein-coding genes (3–5). In particular, neoantigens as tumor-specific private antigens were derived from mutated proteins.

Recent evidence highlights the importance of non-canonical epitopes, which can also elicit anti-tumor immune responses and mainly originated from translation products of transcripts from putative non-protein-coding regions, such as from long non-coding RNAs (lncRNAs), pseudogenes and untranslated regions (UTRs) of coding genes (6–9). Although ∼2% of the entire human genome comprises protein-coding genes, 75% of the rest of the human genome can be transcribed and theoretically translated, potentially offering a pool of previously unexplored peptide targets (10,11). An increasing number of studies suggest that non-canonical epitopes have the potential of being both tumor specific and common among patients, and thereby represent attractive targets for immunotherapy. However, the overall atlas of HLA-bound epitopes from non-coding regions in cancer remains unclear, as does their diversity and tissue specificity across healthy and cancer tissues.

To date, mass spectrometry (MS) has enabled direct identification and quantification of all HLA-presented peptides, defined as the immunopeptidome (2). Several scoring algorithms have been widely used to identify epitopes mapped to the standard, available reference protein databases; however, querying non-canonical epitopes was more challenging because of the lack of non-canonical ORF (ncORF) reference space. Thanks to the accelerating advances in ribosome sequencing (Ribo-seq), thousands of actively translated ncORFs have been pinpointed on the genome. Thus, combining Ribo-seq and MS analysis techniques enables the construction of a comprehensive epitope resource from ncORFs and expands the immunopeptidome in cancer.

On the other hand, the MS data of the HLA immunopeptidome are scattered among innumerable published articles, which is inconvenient for researchers exploring novel candidates for immunotherapy. Currently, several databases have been developed for a canonical immunopeptidome derived from proteins, such as IEDB 3.0 (12), HLA Ligand Atlas (13), T-CoV (14) and DRAMP 3.0 (15). Although valuable information is provided by these databases, at present a specific resource for the systematic study of non-canonical epitopes is lacking. Therefore, the development of an integrated database dedicated to systematic identification and visualization of epitopes encoded by non-coding regions is urgently needed.

**Figure 1.**
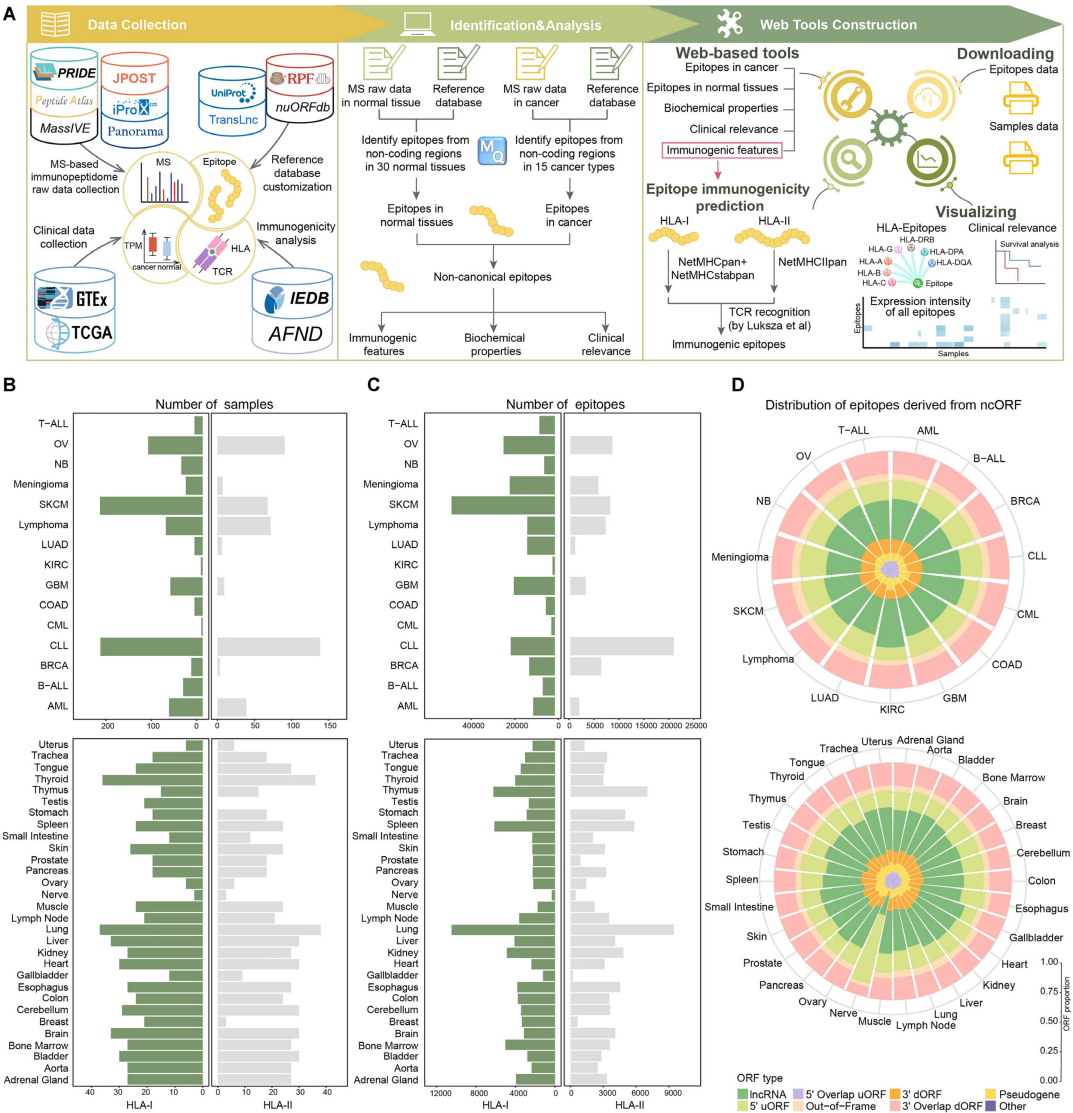
Schematic of the overall design of IEAtlas. (**A**) Data content collection, identification and analysis of non-canonical epitopes, and construction of web tools of IEAtlas. (**B**) Number of cancer and normal samples. (**C**) Number of epitopes identified in cancer and normal tissues. (**D**) Distribution of epitopes derived from ncORFs. Top: cancers; bottom: normal tissues.

In this study, we present IEAtlas (http://bio-bigdata.hrbmu.edu.cn/IEAtlas), a novel database that allows the exploration and visualization of the atlas of HLA-presented immune epitopes derived from non-coding regions (Figure 1A). We manually curated available raw data of the public MS-based HLA immunopeptidome from multiple proteome databases and published studies. We also constructed a theoretical library of peptides encoded by non-coding regions by integrating information on several benchmarked ncORFs. Based on HLA immunopeptidome MS analysis, IEAtlas comprises to date the most comprehensive atlas of non-canonical epitopes bound by HLA-I and HLA-II allotypes among multiple cancer types and non-cancerous tissues. Moreover, their underlying features and relationships with cancer were further investigated, such as biochemical properties, genomic localizations of corresponding ncORFs, immunogenic features as well as cancer specificity. In addition, the clinical relevance of corresponding genes was also analyzed based on their RNA expression levels in thousands of patients across major cancer types and normal tissues.

## MATERIALS AND METHODS

### Constructed theory library of peptides encoded by non-coding regions

We integrated Ribo-seq-supported ORFs from RPFdb (16), nuORFdb (7) and Translnc (17) with their basic annotations. All the ncORFs derived from non-coding regions were filtered, including lncRNAs, 5' upstream ORFs (uORFs), 5' overlap uORFs, out-of-frame ORFs, 3' downstream ORFs (dORFs), 3' overlap dORFs, pseudogenes and others. According to the genome coordinates of ncORFs and the corresponding annotation files, the sequences of ncORFs were obtained by the ‘getblast’ function in the R ‘bedtoolsr’ package using the default parameters. All ncORFs with NTG start codons and TAA/TGA/TAG stop codons were retained. To detect all peptides encoded by ncORFs, all sequences were translated into amino acid sequences. FASTA files of peptides were merged, and peptides entirely contained within other peptides were removed. Only ncORFs that produced peptides >8 amino acids were retained. In total, 475 782 ncORFs were integrated into benchmarked non-canonical peptides for further analysis (Supplementary Figure S1).

### Collection of HLA-MS data

The MS-based immunopeptidome enabled us to directly discover and quantify the entire HLA-presented immunopeptidome, including epitopes derived from non-coding regions (2). All the available MS-based immunopeptidome datasets were obtained from commonly used proteome databases, including PRIDE (18), MassIVE.quant (19), PeptideAtlas (20), JPOST (21), Panorama (22) and iProX (23). Currently, IEAtlas has reanalyzed 2747 samples of 15 cancer types and 30 non-cancerous tissues against the integrated benchmarked theory library of non-canonical peptides.

### Identification of epitopes derived from non-coding regions

The RAW MS data files were downloaded and analyzed by MaxQuant (v.2.1.0.0) (24). Files were searched against both our integrated benchmarked ncORF library and the canonical human proteome obtained from the UniProt database with Swiss-Prot protein evidence (downloaded in February 2022) (25). The maximum allowed precursor mass tolerance was 20 ppm. Modification was set to N-terminal acetylation and methionine oxidation as variable modifications and without fixed modifications (9,13,26). Protease specificity was set to ‘unspecific’. Possible peptide identifications were restricted from 8 to 12 amino acids for HLA-I and from 8 to 25 amino acids for HLA-II. Maximum peptide mass was set to 1500 Da for HLA-I and 3000 Da for HLA-II (9,13). A peptide spectrum match false discovery rate (FDR) of 0.05 was used, and no protein FDR was set; the ‘match between runs’ option was set with default settings and LFQ was set to a ‘minimum ratio count’ of 1. Only epitopes derived from non-coding regions were retained.

### Immunogenic features of epitopes

According to previous studies, presentation and recognition features were used to assess the immunogenicity of non-canonical epitopes via three key parameters, i.e. major histocompatibilty complex (MHC) binding affinity, MHC binding stability and T-cell recognition probability (27,28).

In terms of presentation features, binding affinity was predicted by NetMHCpan (v.4.1) for HLA-I and by NetMHCIIpan (v.4.0) for HLA-II with default settings (Figure 1A) (29). Strong binders were reported as those with a ≤50 nM binding affinity and weak binders were reported as those with a binding affinity of >50 nM and ≤500 nM (9,30). In addition, binding stability was calculated by NetMHCStabpan (v.1.0) for HLA-I using default parameters (31). An expected duration of HLA–epitope interaction >1.4 h was considered to show that the binding of epitopes to HLA was sufficiently stable to enable the presentation of the HLA–epitope complex on the cell membrane through a series of transport processes (27).

In addition, the recognition features were assessed by the multistate thermodynamic model (32). We hypothesized that if a non-canonical epitope is homologous to known pathogen-derived (viral) antigens, it would tend to be recognized by the human T-cell receptor (TCR) repertoire (27,28,33). For each non-canonical epitope, we first obtained sequence alignment scores to human infectious disease-derived, class-I-restricted peptide sequences with positive immune assays from the Immune Epitope Database (IEDB) (downloaded in May 2022) (12). Then, the probability of TCR recognition was inferred by using the model based on alignment scores. An epitope is more likely to be recognized by TCRs if its probability of TCR recognition is high. Therefore, epitopes with MHC binding affinity <500 nM, MHC binding stability >1.4 h and T-cell recognition probability (foreignness) >10^–16^ were defined as immunogenic epitopes.

### Biochemical properties

T-cell reactive epitopes had a significantly higher hydrophobicity score and lower polarity score compared with non-reactive epitopes (34,35). What is more, most of the over-represented and strongly bulky amino acids were also strongly hydrophobic (35). Thus, for biochemical properties of each epitope sequence, we calculated amino acid composition, mean hydrophobicity (Kyte and Doolittle), polarity (Grantham) and bulkiness (Zimmerman) (35–39). In addition, the net charge at pH = 7.0 (Lehninger) and Boman index were also provided for each epitope (40).

### Clinical relevance

Tumor-specific antigens (TSAs) as personalized immunotherapy targets could generate an efficient and safe antitumor immune response as long as they are not expressed in normal tissues (41). The TSA candidates were defined as those epitopes which were only identified in cancer types but not in non-cancerous tissues, or whose corresponding genes were only expressed in cancer by analyzing 11 057 patients across 33 cancer types from TCGA (http://cancergenome.nih.gov/) and 17 382 samples across 30 normal tissues from GTEx (42). Differentially expressed genes were also identified between cancer and normal samples by limma with *P*-values <0.05 (43). Moreover, patients were classified based on the median expression of genes, and the difference in survival between low and high expression groups was compared by log-rank test.

### Database implementation

The server backend of IEAtlas was constructed and accessed based on Java Server Pages with Tomcat container (v.6.0). The MySQL database (v.5.5.48) was used to document and manage all the metadata in IEAtlas. HTML, JavaScript and CSS code constituted the web frontend of IEAtlas. The visualization of all analysis results and multiple statistical tables was supported by jQuery (v.3.3.1), Datatable (v.1.10.25) and ECharts (v.5.5.1) plugins. The R framework (v.3.6.3) was performed for statistical analyses. IEAtlas has been tested on several popular web browsers, including Google Chrome (preferred), Firefox and Apple Safari browsers.

## DATABASE CONTENT

Currently, IEAtlas has re-analyzed public MS-based immunopeptidome datasets from 1444 cancer samples covering 15 cancer types and 1303 non-cancerous samples of 30 tissues (Figure 1B). Based on data collection and integrative analysis, there were 174 465 and 94 375 non-canonical epitopes detected from cancer and normal tissues, respectively, of which 60.60% and 41.90% non-canonical epitopes were bound by HLA-I and HLA-II allotypes, respectively (Figure 1C). These epitopes mainly originated from lncRNAs, 5' uORFs and 3' overlap dORFs in cancer or normal tissues (Figure 1D). Next, by analyzing the length distribution, we found that the abundance of 9-mers was the highest for HLA-I epitopes, while 13–18-mer epitopes were frequently presented by HLA-II molecules (Supplementary Figure S2), which is consistent with previous studies (13,44). Importantly, there were 54 017 epitopes for HLA-I and 51 015 epitopes for HLA-II passing HLA immunogenic tests, which account for 37.16% and 50.76% of HLA-I- and HLA-II-binding epitopes, respectively.

To provide a convenient way for users to investigate the functions of epitopes and genes of interest, IEAtlas has also developed five flexible tools. Considering the strong tumor/tissue specificity of epitopes, IEAtlas allows users to access each epitope entry in cancer or normal tissues of interest by quick searching or query options. In addition, multiple visualization results are offered for facilitating comprehension of their roles in cancer immunity.

## WEB INTERFACE

**Figure 2.**
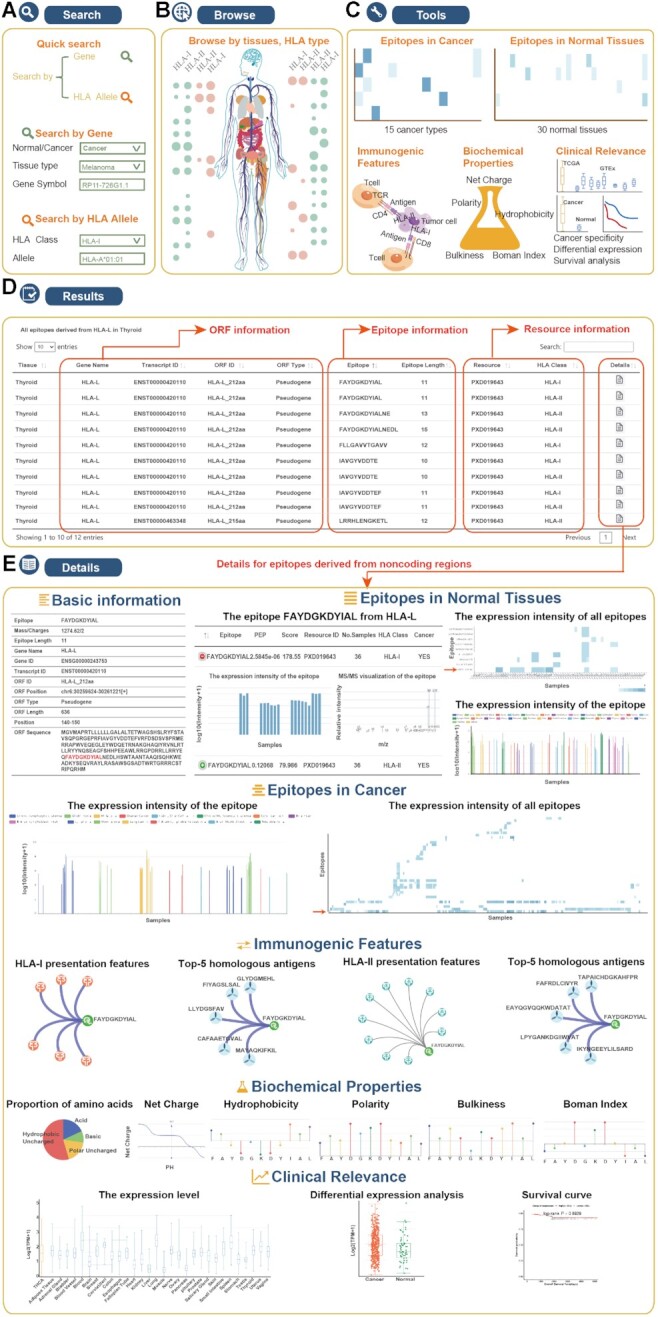
User interface and workflow of using IEAtlas. (**A**) Search page for non-canonical epitopes derived from genes or HLA allele of interest. (**B**) Browse page for non-canonical epitopes by tissues or HLA type. (**C**) Interactive tools provided in IEAtlas. (**D**) The result list for non-canonical epitopes. (**E**) Detailed information for non-canonical epitopes with diverse immunogenic features, the expression intensity of epitopes in cancer and normal tissues, HLA presentation features, top ranked homologous antigens, biochemical properties and clinical relevance in diverse cancer types are also provided in IEAtlas resource.

IEAtlas allows users to explore the comprehensive atlas of HLA-presented immunogenic epitopes derived from non-coding regions, and further identify the potential immunotherapy targets. The user-friendly web interface of IEAtlas allows users to query, browse, visualize and download data (Figure 2). On the ‘Search’ page, IEAtlas allows users to query genes encoding epitopes in cancer or normal tissues of interest. In addition, users are also allowed to search epitopes by the specific HLA allele of interest (Figure 2A). On the ‘Browse’ page, the user can browse epitopes bound by HLA-I or HLA-II across different cancer or normal tissues (Figure 2B). Moreover, five types of interactive tools (epitopes in cancer, epitopes in normal tissues, immunogenic features, biochemical properties and clinical relevance) are provided in IEAtlas to retrieve detailed information about non-canonical epitopes from different aspects (Figure 2C). On the ‘Result’ page, genes and corresponding encoded epitope information, resources and MHC types are shown (Figure 2D).

Entering the detailed page for each epitope, the users can obtain extensive information about that epitope and the other epitopes produced by the same parent gene (Figure 2E). First, the basic information for the epitope is provided in a table. Considering the strong tumor/tissue specificity of epitopes, not only is the expression intensity of the epitope in all cancer types and normal tissues provided but also that for the other epitopes derived from the same gene. Next, the binding interactions between epitopes and HLA alleles are demonstrated as a network. Similarly, according to the sequence alignment scores, the top five homologous antigens in IEDB are also shown as a network. Users can click the corresponding icons to enter the AFND or IEDB database (12,45). In addition, the biochemical properties of epitopes are provided in table and figure forms. IEAtlas allows users to compare gene expression across tissues and diverse cancer types, and the difference in survival between low and high expression groups. Multiple visualization results are provided for promoting understanding of their roles in cancer. IEAtlas supports the download of the identified epitopes, basic information, biochemical properties and sample information on the ‘Download’ page and a detailed tutorial is provided on the ‘Help’ page for users to understand how to use IEAtlas.

## EXAMPLE APPLICATION

**Figure 3.**
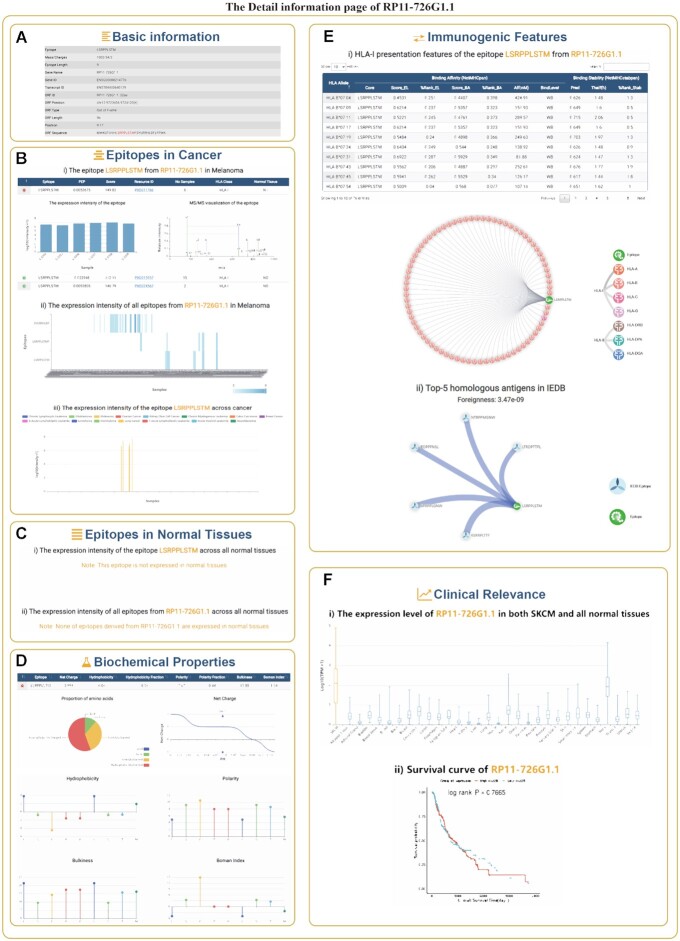
The detailed information page of RP11-726G1.1 in IEAtlas. (**A**) Basic information on RP11-726G1.1. (**B**) Epitopes from RP11-726G1.1 in cancer. (**C**) Epitopes from RP11-726G1.1 in normal tissues. (**D**) Biochemical properties of epitopes from RP11-726G1.1. (**E**) Immunogenic features of epitopes from RP11-726G1.1. (**F**) Clinical relevance of RP11-726G1.1 in cancer.

An epitope derived from the pseudogene RP11-726G1.1 has been experimentally confirmed to bind HLA alleles *in vitro* in melanoma (7). When querying the epitopes derived from RP11-726G1.1 by ‘Gene Search’ in melanoma, we obtained three epitopes supported by four resources (Figure 3). These epitopes were melanoma specific and shared among tumor patients. Moreover, both the epitopes ‘LSRPPLSTM’ and ‘SYLRRHLDF’ were predicted to be presented by HLA-I alleles and recognized by TCRs. In addition, these epitopes show positive net charges, similar to the feature of antimicrobial peptides. Based on the RNA expression level, RP11-726G1.1 was only highly expressed in skin cutaneous melanoma (SKCM) and testis tissue, suggesting that RP11-726G1.1 may be a cancer testis antigen. Another example is the gene MAGEA11, a member of the melanoma cancer testis antigen family, which is epigenetically silenced in most normal adult tissues, except germ and placental trophoblast cells (1). When querying epitopes derived from MAGEA11 in melanoma (Supplementary Figure S3), we obtained six entries. Indeed, we found that the majority of epitopes derived from MAGEA11 were only expressed in melanoma samples. Consistent with previous studies, the parent gene of epitopes does not express MHC protein and is thereby ‘invisible’ to the immune system. Moreover, based on the RNA expression of MAGEA11 across SKCM and normal tissues, we found that the expression level of MAGEA11 in the testis far exceeds that of other tissues, and high expression of MAGEA11 was associated with worse survival of cancer patients (Supplementary Figure S3). In addition, we further discovered many epitopes from non-coding regions which only exist in cancer, and have potential for therapeutic cancer vaccines (Supplementary Figure S4).

## CONCLUSIONS AND FUTURE DEVELOPMENT

Checkpoint blockade therapy has changed the paradigm of cancer treatment, but specific antigen targets remain largely uncharacterized. Integration of non-canonical tumor epitopes into current state-of-the-art immunotherapy strategies would expand the armamentarium of immunotherapy options available (46). By systematic analysis of HLA immunopeptidome MS data against our integrated benchmarked ncORF information, the IEAtlas database provides a comprehensive epitope atlas derived from non-coding regions, including thousands of non-canonical epitopes presented by HLA-I and HLA-II. These epitopes mainly originated from lncRNAs, 5' uORFs, 3' overlap dORFs, out-of-frame ORFs, and so on. It is noteworthy that the immunogenicity evaluation of each epitope in IEAtlas was based on the commonly used pipelines, including both the experimental and prediction results of HLA presentation and TCR recognition. Among the atlas of epitopes in IEAtals, several tumor-specific epitopes indeed have been validated in previous studies, such as RP11-726G1.1 and MAGEA11.

IEAtlas is an important resource that focuses on HLA-presented immune epitopes derived from non-coding regions, and multiple visualization results were provided to facilitate understanding their roles in cancer immunity. Considering the strong tumor/tissue specificity of epitopes, IEAtlas allows users to access each epitope entry in cancer or normal tissues of interest by quick searching or query options. Moreover, IEAtlas also integrated useful tools for investigating their tissue- and cancer-specific expression patterns, immunogenic features, biochemical properties and clinical association. Thus, IEAtlas provides a one-stop service for users to explore the important roles of non-canonical epitopes in cancer immunotherapies.

Moreover, there is still room for improvement of IEAtlas. In the future, we will continue to update and integrate the data content in IEAtlas by: (i) analysing the available datasets of the HLA immunopeptidome to expand the current atlas of non-canonical epitopes; (ii) constructing a more general resource for ncORF regions by integrating newly generated omic datasets about translation; and (iii) incorporating the information on experimentally defined T-cell epitopes. These additions are anticipated to enhance the efficiency of IEAtlas and it will be an important database for investigating the immunogenic capacity of non-canonical epitopes and their potential as therapeutic cancer vaccines. More rigorous investigation of these non-canonical immune epitopes would lead to a profound understanding and provide new insights into cancer immunotherapy, which should be prioritized in future perturbation studies.

## DATA AVAILABILITY

IEAtlas is an open resource for HLA-presented immune epitopes derived from non-coding regions (http://bio-bigdata.hrbmu.edu.cn/IEAtlas).

## Supplementary Material

gkac776_Supplemental_File
