# Supplementary material for: IEAtlas: an atlas of HLA-presented immune epitopes derived from non-coding regions
Source: Nucleic Acids Res. 2022 Sep 13;51(D1):D409–17. doi: 10.1093/nar/gkac776 (PMC9825419; doi:10.1093/nar/gkac776)
Supplement: gkac776_Supplemental_File [file gkac776_supplemental_file.docx]

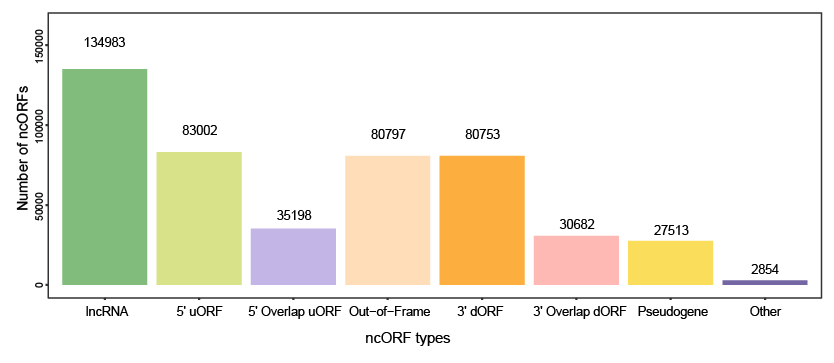
**Figure S1. Number of ncORFs for diverse types.**


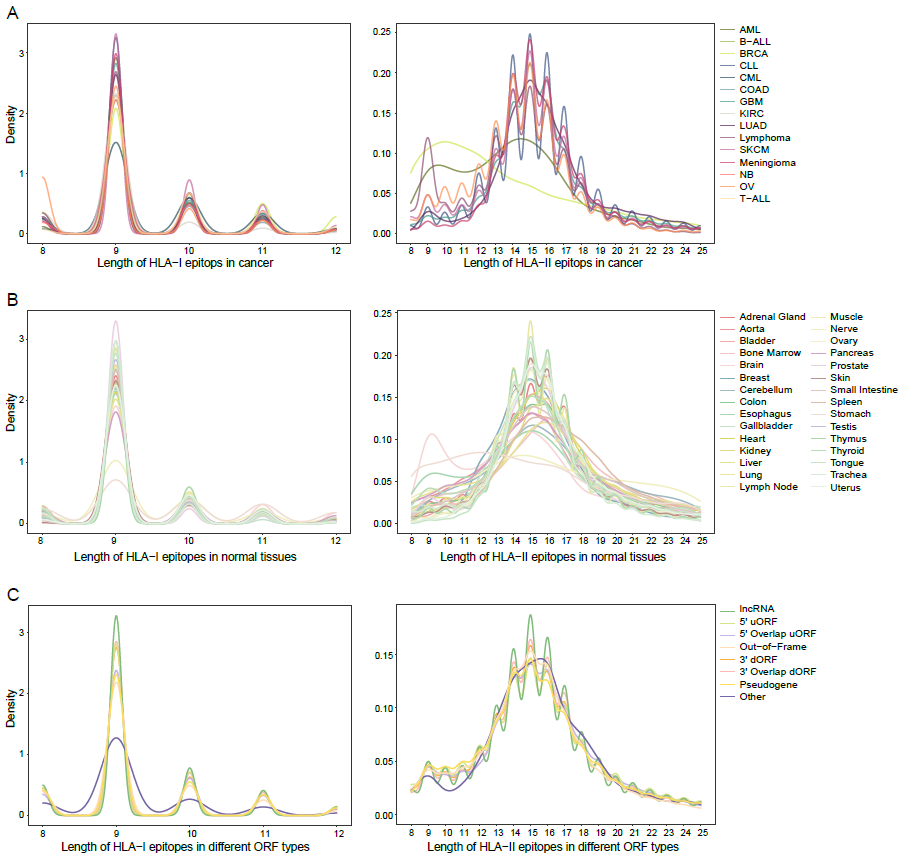


**Figure S2. Density of different length of HLA-I or HLA-II epitopes for different ORF types in cancer and normal tissues.** (A) for different cancer types; (B) for normal tissues and (C) for different ORF types.


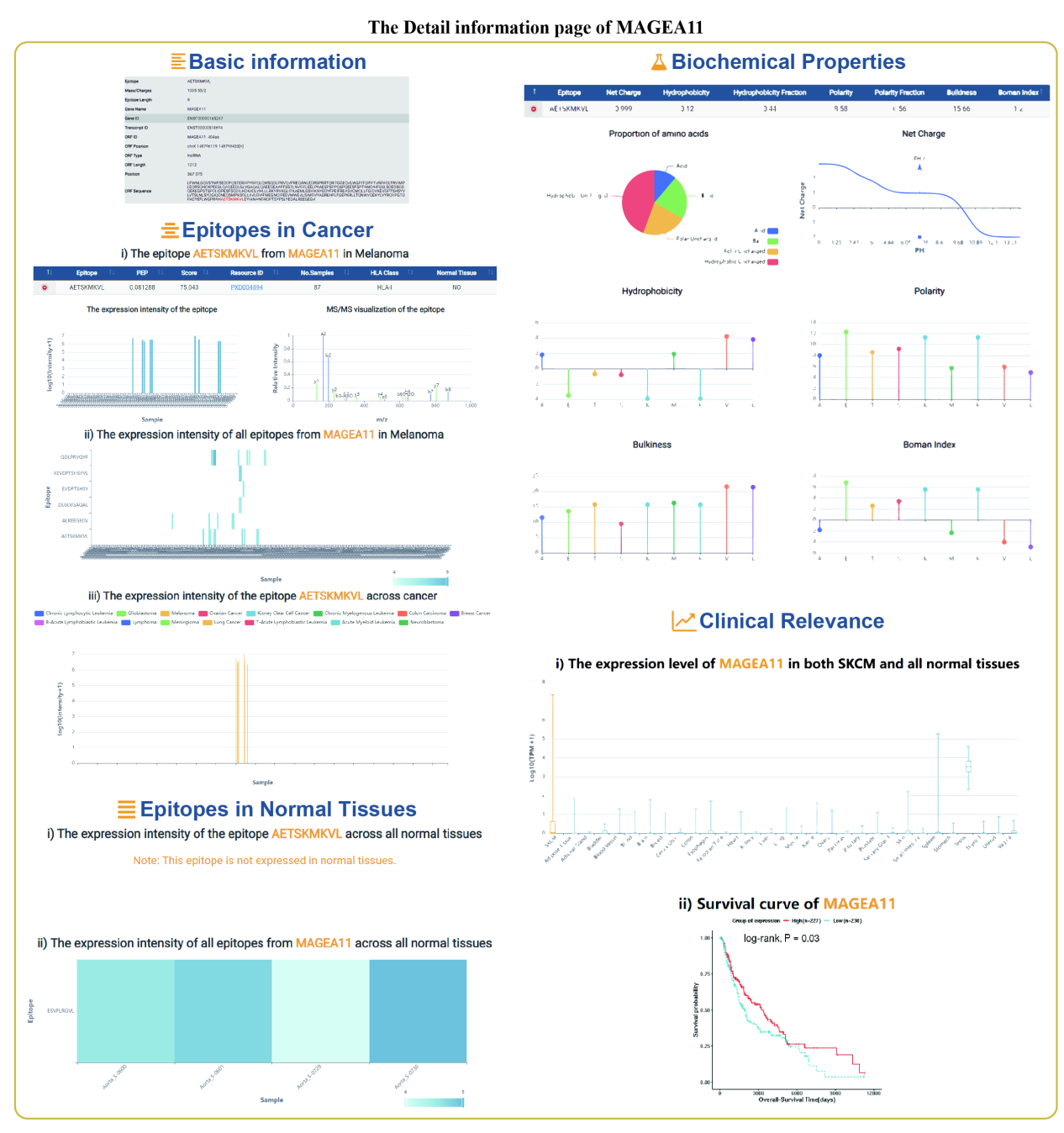


**Figure S3. The Detail information page of MAGEA11 in IEAtlas.**


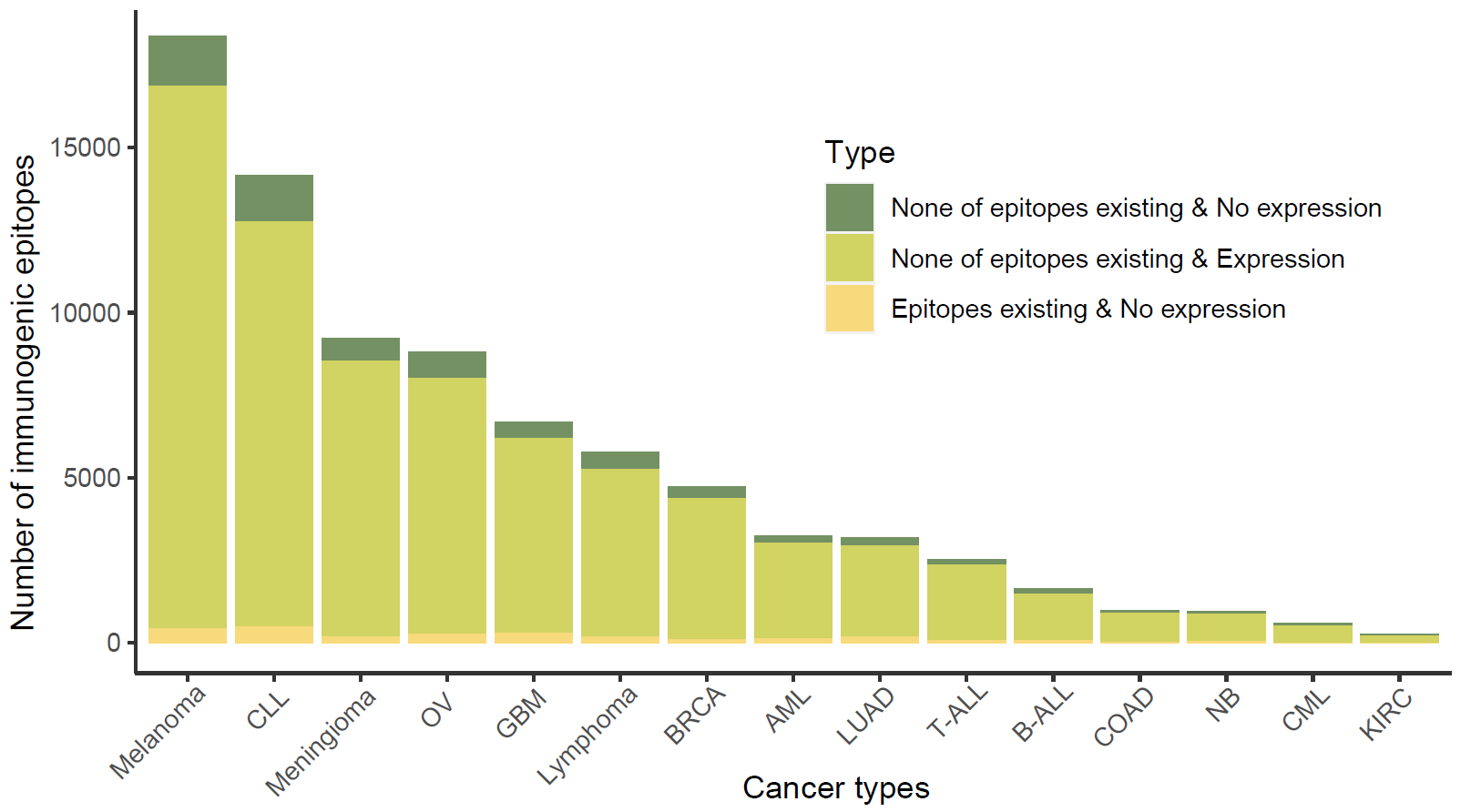


**Figure S4. Number of immunogenic epitopes across different cancer types.**
